# Abnormal lipid droplets accumulation induced cognitive deficits in obstructive sleep apnea syndrome mice via JNK/SREBP/ACC pathway but not through PDP1/PDC pathway

**DOI:** 10.1186/s10020-021-00427-8

**Published:** 2022-01-14

**Authors:** Dongze Li, Na Xu, Yanyan Hou, Wenjing Ren, Na Zhang, Xi Wang, Yeying Sun, Wenxue Lu, Guiwu Qu, Yan Yu, Changjun Lv, Fang Han

**Affiliations:** 1 Binzhou Medical University, 346 Guanhai Road, YanTai, 264003 China; 2 Yantai Affiliated Hospital of Binzhou Medical University, 717 Jinbu Street, YanTai, 264199 China

**Keywords:** Chronic intermittent hypoxia, Cognitive deficits, ROS, Lipid droplets, JNK/SREBP/ACC pathway

## Abstract

The mechanisms of chronic intermittent hypoxia (CIH)-induced cognitive deficits remain unclear. Here, our study found that about 3 months CIH treatment induced lipid droplets (LDs) accumulation in hippocampal nerve and glia cells of C57BL/6 mice, and caused severe neuro damage including neuron lesions, neuroblast (NB) apoptosis and abnormal glial activation. Studies have shown that the neuronal metabolism disorders might contribute to the CIH induced-hippocampal impairment. Mechanistically, the results showed that pyruvate dehydrogenase complex E1ɑ subunit (PDHA1) and the pyruvate dehydrogenase complex (PDC) activator pyruvate dehydrogenase phosphatase 1 (PDP1) did not noticeable change after intermittent hypoxia. Consistent with those results, the level of Acetyl-CoA in hippocampus did not significantly change after CIH exposure. Interestingly, we found that CIH produced large quantities of ROS, which activated the JNK/SREBP/ACC pathway in nerve and glia cells. ACC catalyzed the carboxylation of Acetyl-CoA to malonyl-CoA and then more lipid acids were synthesized, which finally caused aberrant LDs accumulation. Therefore, the JNK/SREBP/ACC pathway played a crucial role in the cognitive deficits caused by LDs accumulation after CIH exposure. Additionally, LDs were peroxidized by the high level of ROS under CIH conditions. Together, lipid metabolic disorders contributed to nerve and glia cells damage, which ultimately caused behavioral dysfunction. An active component of *Salvia miltiorrhiza*, SMND-309, dramatically alleviated these injuries and improved cognitive deficits of CIH mice.

**Supplementary Information:**

The online version contains supplementary material available at 10.1186/s10020-021-00427-8.

## Introduction

Chronic intermittent hypoxia (CIH) is the main feature of obstructive sleep apnea syndrome (OSAS). In addition to hyperlipidemia, atherosclerosis, and high cardiovascular risk (Hu et al. 2021), CIH can also cause serious neurocognitive dysfunction, which is associated with regional alterations in hippocampus morphology (Zhang et al. 2020). Currently, lipid droplets (LDs) accumulation has been referred to neurodegeneration (Farmer et al. 2020), such as hereditary spastic paraplegia (HSP) (Inloes et al. 2018), Parkinson's disease (Han et al. 2018) and Alzheimer’s disease (Hamilton et al. 2015). Nevertheless, the role of LDs accumulation in CIH-induced neuro dysfunction still needs to be determined. Under physiological conditions, the irreversible oxidative decarboxylation plays a central role in lipid homeostasis. Dephosphorylation of pyruvate dehydrogenase phosphatase 1 (PDP1) boosts the acetylation status of pyruvate dehydrogenase complex E1ɑ subunit (PDHA1) and PDP1, which contributes to the subsequent pyruvate dehydrogenase complex (PDC) activation (Fan et al. 2014). Then, activated PDC converts pyruvate to Acetyl-CoA (Walther et al. 2017). As an important molecule in the metabolism processes of the human body, Acetyl-CoA is the raw material for de novo lipogenesis (Walther et al. 2017). ACC, the rate-controlling enzyme in the pathway of lipogenesis, catalyzes the carboxylation of acetyl-CoA to malonyl-CoA (a highly regulated molecule in fatty acid synthesis) (Hunkeler et al. 2018). Therefore, PDP1/PDC/ACC played a crucial role in dyslipidemia. As the production of CIH, ROS is closely related to variety of physiological processes including lipid metabolism (Prabhakar et al. 2012). Abnormal accumulation of ROS could regulate the expression of genes implicated in lipid metabolism, including SREBP, ACC, CPT-1, SCD-1 (Zhao et al. 2018). However, the potential mechanisms under abnormal lipid metabolism and its functional implication in diseases remain unknown.

SMND-309 is a major potent component extracted from *salvia miltiorrhiza*. It can be detected in the rat brain as a novel metabolite of salvianolic acid B after administration (Tian et al. 2009). Research has proven that SMND-309 promotes neuron survival through antiapoptotic, anti-inflammatory and antioxidative effects (Yang et al. 2010). In this study, SMND-309 was intraperitoneally injected into mice to alleviate CIH-induced neuro damage. This might be helpful to develop a potential drug therapy target for OSAS.

## Material and methods

### Antibodies and reagents

A rabbit anti-c-Jun N-terminal kinase (JNK) antibody (4668, CST), rabbit anti-phospho-JNK antibody (9252, CST), rabbit anti-Acetyl-CoA carboxylase (ACC) antibody (3676, CST), rabbit anti-NeuN antibody (24037, CST) and rabbit anti-cleaved caspase 3 (9664, CST), rabbit anti-PDP1 antibody (D8Y6L, CST) were purchased from Cell Signaling Technology (Massachusetts, USA). A rabbit anti-sterol regulatory element-binding protein (SREBP) antibody (ab28481, Abcam), rabbit anti-Iba-1 antibody (ab178847, Abcam), goat anti-glial fibrillary acidic protein (GFAP) antibody (ab53554, Abcam), mouse anti-doublecortin (DCX) antibody (ab135349, Abcam), rabbit anti-β-actin antibody (ab8227, Abcam), horseradish peroxidase-conjugated AffiniPure goat anti-rabbit IgG (H + L) (ab7090, Abcam), Alexa Fluor 647 AffiniPure donkey anti-rabbit IgG (H + L) (ab150063, Abcam), Alexa Fluor 647 AffiniPure donkey anti-mouse IgG (H + L) (ab150111, Abcam), Alexa Fluor 594 AffiniPure donkey anti-rabbit IgG (H + L) (ab150064, Abcam), Alexa Fluor 594 AffiniPure donkey anti-goat IgG (H + L) (ab150136, Abcam) and Alexa Fluor 555 AffiniPure donkey anti-rabbit IgG (H + L) (ab150066, Abcam) were all obtained from Abcam (Cambridge, UK). A rabbit anti-p-PDHA1 antibody (ABS204m, Millipore) was from Millipore. Nile Red (72485) was purchased from Sigma-Aldrich. A rabbit anti-PDHA1 antibody (MA5-32545, Thermo), BODIPY (493/503) (D3922, Thermo Fisher) and C11-BODIPY (581/591) (D3861, Thermo Fisher) were from Thermo Fisher. SMND-309 (molecular formula C_18_H_14_O_8_, molecular weight 358.3) was provided by professor Guiwu Qu. The SMND-309 powder was dissolved in the normal saline, and the test mice were intraperitoneally injected at 25 mg/kg of body weight (Tian et al. 2009) (Zhu et al. 2013). 3-fluoropyruvate (3-FP, F4004, Sigma), a competitive inhibitor of PDHA1, was dissolved in PBS, and intraperitoneal injections of the 3-FP every 3 days at 80 mg/kg for 1 months (Chen et al. 2018). In vitro experiment, the cells were added 3-FP to make the final concentration in the culture medium was 1uM after planted for 12 h.

### Animals

Male C57BL/6 mice have been used to study the damage of OSAS and the potential molecular and pathological mechanisms in many studies. Six -week-old male wild-type (WT) mice on a *C57BL/6* background (Jinan Pengyue Experimental Animal Breeding Co., Ltd., China) were used for this study. All mice were maintained at 23 ± 1 °C with a 12 h/12 h light/dark cycle. Food and water were available ad libitum. Before the tests, 40 mice were randomly divided into 4 groups (WT + RA, WT + CIH, SMND-309, SMND-309 + CIH)/(WT + RA, WT + CIH, 3-FP, 3-FP + CIH), and each group had 10 mice. Every mouse was given a unique number. During the whole testing process, the researchers were blinded to the treatment. All the mice were fed and used according to the NIH guidelines, and this study was approved by the Ethics Committees on Animal Experimentation of Binzhou Medical University (Permit No. SCXK20160006).

### Cell culture

The neuron cell line HT22 (JNO-02001) was purchased from Guangzhou Jinio Biotechnology Co., Ltd. The cell was cultured with DMEM medium containing 10% FBS, under standard incubation conditions at 37 °C, 5% CO_2_. Cell culture medium was replaced every 3 days and cells were passaged when they reached 80–90% confluency. The HT22 cells were seeded onto cover slips and randomly divided into four groups: normal control (NC) group (n = 4; RA exposure), intermittent hypoxia (IH) group (n = 4; IH exposure), NC + 3-FP group (n = 4; RA exposure and 3-FP treatment), and IH + 3-FP group (n = 4; IH exposure and 3-FP treatment).

### Low oxygen exposure

#### CIH exposure

The mice were exposed to CIH/room air (RA) conditions from 8:00 AM–4:00 PM per day for about 3 months. During the exposure, the mice in the WT + CIH group and the SMND-309 + CIH (3-FP + CIH) group were placed into the CIH chamber (BioSpherix OxyClycler A84, USA), the WT + RA group and the SMND-309 (3-FP) group were placed into the RA chamber (BioSpherix OxyClycler A84, USA). The CIH program was performed as described previously (Li et al. 2020). Firstly, the chamber was filled with N_2_ for 85–95 s, and the oxygen level was reduced from 21% ± 1% (normal) to 7% ± 1% (hypoxia). The oxygen level was then maintained at 7% ± 1% for 15–20 s. Finally, the level was recovered to 21% ± 1% in 45–50 s and sustained for 15–20 s. A cycle of CIH lasted approximately 180 s, and there were approximately 20 cycles per hour. The oxygen level in the RA chamber was always maintained at 21% ± 1%.

#### IH exposure

IH was induced by culturing cells in an oxygen control cabinet (Biospherix Oxycycler C42) mounted within an incubator and equipped with oxygen sensor for continuous oxygen level monitoring. A mixture of nitrogen oxygen and 5% CO_2_ was infused and oxygen levels consisted of a 30-min hypoxic period (3% O2), followed by 30 min of reoxygenation (21% O_2_). Actual O_2_ saturation was kept at 3% for 10 min, at each 1-h cycle (Dyugovskaya et al. 2012). This IH treatment was performed for 3 day.

### Behavioral testing

#### Three-chamber social test

To examine cognitive function impairment due to CIH treatment, the three-chamber social test, Morris water maze (MWM) test and fear conditioning test were performed. The three-chamber social test was conducted as previously described (Moy et al. 2004). The chamber used in the test (60 cm L × 26 cm W × 30 cm H) was divided into three sections: center, left and right (20 cm L × 26 cm W). The test mouse had free access to the different sides of the chamber through the door (11 cm L × 11 cm W) on the dividing wall. The test consisted of 3 phases with 20 min intervals. Each phase allowed the test mouse to explore the chamber for 10 min. During the phase, the video system tracked the behavior of the mouse. At phase 1, a test mouse was placed in the center chamber, and two empty wire cages (10 cm D × 14 cm H) were placed into the left and right sides. During phase 2, an unfamiliar (stranger) mouse was placed in the left wire cage, and an empty wire cage was placed on the right side. Another stranger mouse was then placed in the right wire cage in phase 3. Mice were tested from approximately 8:00 AM to 12:00 AM and received CIH/RA treatment from 12:00 AM to 4:30 PM.

#### MWM

The MWM test was conducted as previously described (Lee et al. 2019). The system contained a circular pool (122 cm D × 51 cm H) and an escape platform (12 cm D × 34 cm H) (ZS Dichuang, Beijing, China). The pool was divided into four equal quadrants and filled with 35 cm of deep water. The water was dyed with TiO_2_ and kept at 23 ± 1 ℃. The escape platform was positioned 1 cm below the water surface in quadrant two. The same discriminate landmarks were placed around the maze during the test. The test consisted of two stages, including four days of the training stage and one day of the probe trial stage.

The day before the test, all the mice were acclimated to the pool without the platform for 60 s. During the training stage, the test mouse performed four trials for 4 consecutive days. In the first trial, a mouse was put into the water facing the pool wall at quadrant one. If the mouse found the escape platform in 60 s, the tracker system would be automatically closed; otherwise, the mouse would be guided to the platform and forced to stay on it for 15 s. With a 15 min interval, the mouse received a second trial and started at quadrant two. After trials 3 and 4, the mouse was dried with a towel and placed back into the home cage. At the probe trial stage (day 5), the mouse was put into the water at quadrant four and forced to swim without the platform for 60 s. Several performance parameters of the test mouse were recorded, including the total swimming distance, the number of platform crossings, the duration and the distance traveled in the target quadrant. The tests were performed from 8:00 AM to 12:00 AM. After the tests, the mice were exposed to CIH/RA conditions from 12:00 AM to 4:30 PM.

#### Fear conditioning test

The equipment consisted of a conditioning chamber with a sound-attenuating wall and a video monitoring system (ZS Dichuang, Beijing, China). An acrylic box (35 cm L × 35 cm W × 35 cm H) and a stainless-steel grid floor were placed in the chamber. The floor was connected to a device to deliver the footshock, and tests were performed as previously described (Shoji et al. 2014). On day 1, after acclimating the chamber (black acrylic box with a jasmine smell) for 2 min, the test mouse underwent 30 s of sound (65 dB, 3 kHz) three times (separated by 30-s intervals), and the mouse received a foot shock (0.7 mA) during the last 2 s of each sound. On day 2, the mice were exposed to the same environment (black acrylic box with a jasmine smell) for 5 min with no sound and no foot shock. The fear behavior (freezing) of the mouse, which referred to contextual memory, was recorded. On day 3, after 2 min of acclimatization to the new environment (blue acrylic box with a lemon smell), the mouse was exposed to 30 s of sound (65 dB, 3 kHz) three times (separated by 30-s intervals). During this stage, the fear behavior (freezing), which refers to cued memory, was measured.

### Tissue preparation

Five mice from each group were deeply anesthetized by 4% choral hydrate through intraperitoneal injection. They were then perfused with physiological saline via the left ventricle of the heart, and fixed with 4% ice-cold paraformaldehyde. The fixed brains were embedded in optimal cutting temperature compound, frozen in liquid nitrogen and stored at − 80 °C. For the other mice, the hippocampi were harvested and stored in liquid nitrogen.

### Transmission electron microscopy (TEM)

The hippocampi of each group were fixed in 2.5% ice-cold glutaraldehyde in 0.1 M PBS at pH 7.4 and osmium tetroxide. After gradient dehydration with ethyl alcohol and acetone, the hippocampi were embedded in epoxy resin Epon 812. Tissues were then cut into ultrathin sections (50 nm) by a CM1900 microtome (Leica, Germany) and stained with uranyl acetate and lead citrate. Samples were viewed in a JEOL JEM 1010 TEM at 80 kV and captured through an AMT XR-16 mid-mount 16 mega-pixel digital camera (Hu et al. 2011).

### Hematoxylin and eosin (H&E) staining

The brains of each group were cut into 5 μm sections and stained with H&E. After dehydration with ethanol, the sections were observed by an Invitrogen EVOS M5000 imaging system (Thermo Fisher, USA) (Ballok et al. 2004).

### Nile red staining

To detected the accumulation of LDs, the frozen slices were washed three times in PBS and incubated with Nile red (1:1000) for 10 min. After washing three times with PBS, the slices were mounted with Vectashield with DAPI (Vector Labs, USA) and imaged with an Invitrogen EVOS M5000 imaging system (Thermo Fisher, USA). The Nile red powder was dissolved in methyl alcohol at a concentration of 1 mg/mL.

#### Measure the levels of PDC activity, acetyl-CoA and ROS

Pyruvate Dehydrogenase Activity Assay Kit (MAK183) was purchased from Sigma. Acetyl-Coenzyme A Assay Kit (Sigma, MAK183) was used to determine the Acetyl-CoA level. The contents of ROS in the hippocampus were detected with an Aconitase Activity Assay Kit (Sigma-Aldrich, MAK051) (Liu et al. 2015). All the protocols were conducted in accordance with the manufacturer’s instructions. The optical density of the wells was detected by the Ultra Multitask Ascent (Epoch, BioTek, USA).

#### Immunofluorescence

After washing three times with PBS, the frozen slices were permeabilized with 0.3% Triton X-100 (T8200, Solarbio, Beijing, China) in PBS for 5 min. The slices were then incubated with 10% donkey serum (Jackson ImmunoResearch, Pennsylvania, USA) for 30 min at room temperature and were then incubated with primary antibodies including mouse anti-DCX (1:200), rabbit anti-cleaved-caspase 3 (1:200), rabbit anti-Iba-1 (1:200), goat anti-GFAP (1:300) or rabbit anti-NeuN (1:200) overnight at 4 °C. The secondary antibodies were maintained at room temperature for 1 h and included Alexa Fluor 647 AffiniPure donkey anti-rabbit (1:500), Alexa Fluor 647 AffiniPure donkey anti-mouse (1:500), Alexa Fluor 594 AffiniPure donkey anti-rabbit (1:500), Alexa Fluor 594 AffiniPure donkey anti-goat (1:500) or Alexa Fluor 555 AffiniPure donkey anti-rabbit (1:500). After staining with BODIPY (493/503) (1:200) for 15 min or C11-BODIPY (581/591) (1:500) for 30 min and washing the sections three times in PBS, the sections were mounted with Vectashield with DAPI (Vector Labs, USA) and imaged using an Invitrogen EVOS M5000 imaging system (Thermo Fisher, USA) (Liu et al. 2015). The BODIPY (493/503) powder was dissolved in PBS at 1 mg/mL, and the C11-BODIPY (581/591) powder was dissolved in DMSO at 2 mg/mL.

#### Protein extraction and western blot

After suspending the proteins in RIPA lysis buffer (R0020, Solarbio, Beijing, China), the protein concentrations were measured by a bicinchoninic acid assay kit (PC0020, Solarbio, Beijing, China). The samples were then homogenized in SDS loading buffer (9173, TaKaRa, Beijing, China) and separated through SDS-PAGE with 8–12% gels (P0012, Beyotime, Shanghai, China). After being transferred to polyvinylidene difluoride membranes (03010040001, Roche, USA) in an ice bath, the membranes were blocked with 5% nonfat dry milk in TBST at room temperature. They were then incubated with an anti-JNK antibody (1:1000), anti-P-JNK antibody (1:1000), anti-SREBP antibody (1:1000), anti-ACC antibody (1:1000), anti-PDP1 antibody (1:1000), anti-PDHA1 antibody (1:5000), anti-p-PDHA1 antibody (1:2000) or anti-β-actin antibody (1:5000) overnight at 4 °C. After washing three times with TBST, the membranes were incubated with the horseradish peroxidase-conjugated secondary antibody (1:5000) at room temperature for 1 h. Finally, the blots were developed using an electrochemiluminescence detection system (ChemScope 6000, Clinx Science Instruments CO., Ltd., China).

#### RT-PCR

The total RNA was prepared from hippocampal tissue samples or cells using Trizol reagent (Invitrogene) following the manufacturer’s instructions. Isolated total RNA was quantified spectrophotometrically. Aliquots of total RNA were reverse transcribed using cDNA synthesis kit (Takara RR047A) according to the manufacturer’s protocol. PCR primers (PDHA1, FP: 5′-GAAATGTGACCTTCATCGGCT-3′, RP: 5′-TGATCCGCCTTTAGCTCCATC-3′; PDP1, FP: 5′-GCACCCATAGAGGACCGGA-3′, RP: 5′- CCTGCATGACCATCAAAAACCC-3′) were purchased from Thermo Fisher. Standard curves for gene of interest and housekeeping gene (β-actin) were included in each reaction. Using SYBR Green PCR Master Mix to quantify, 40 cycles of RT-PCR were performed on an ABI 7500 thermal cycler (Applied Biosystems).

### Statistical analysis

All experiments were repeated three times. The experimenter was unaware of the animal’s group during experimentation and removed the mice that were in poor condition (they did not like to exercise). SPSS version 24.0 (IBM Crop, Chicago, USA) was used for statistical analysis. Data are displayed as the mean ± SEM. One-way analysis of variance (ANOVA) and a Bonferroni post hoc test were used to evaluate the results of the three-chamber social tests (Xu et al. 2019). The results of escape latency in the MWM test were examined through three-way mixed ANOVA (Champagne et al. 2002). For the total traveling distance, platform crossing counts, the duration and distance in the target zone and the fear conditioning tests were assessed with two-way ANOVA (Yanai and Endo 2021). Other data were assessed with two-way ANOVA. Statistical significance was accepted at p < 0.05.

## Results

### The exposure of CIH

**Fig. 1:**
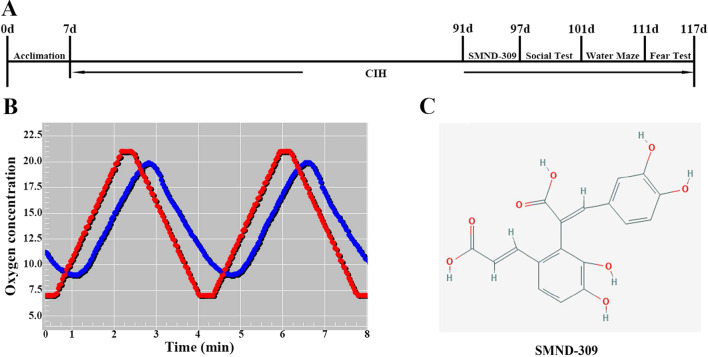
The process of research and the structure of SMND-309. **A** The study workflow. After 7 days of acclimation, the mice were exposed to the CIH condition for about 3 months, and they were treated with SMND-309 at the last week. Behavioral tests were conducted as soon as CIH exposure finished. **B** The CIH treatment program. The red line represents the set point. The blue line represents the actual oxygen level. **C** The structure of SMND-309

The mice were exposed to RA/CIH condition for about 3 months (day7-day97) (shown in Fig. 1B). A week before the behavioral experiments (day91-day97), the mice were intraperitoneal injected with SMND-309 for once daily at 25 mg/kg of body weight. The three-chamber social test, the MWM test and the fear conditioning test were conducted in sequence as soon as the treatment was finished (day97–day117) (shown in Fig. 1A). Due to the long time required for behavioral experiments, mice in the model group should be kept in a hypoxic environment, and samples should be taken immediately after the experiment.

### CIH exposure caused the social damage, SMND-309 could attenuate the damage

**Fig. 2:**
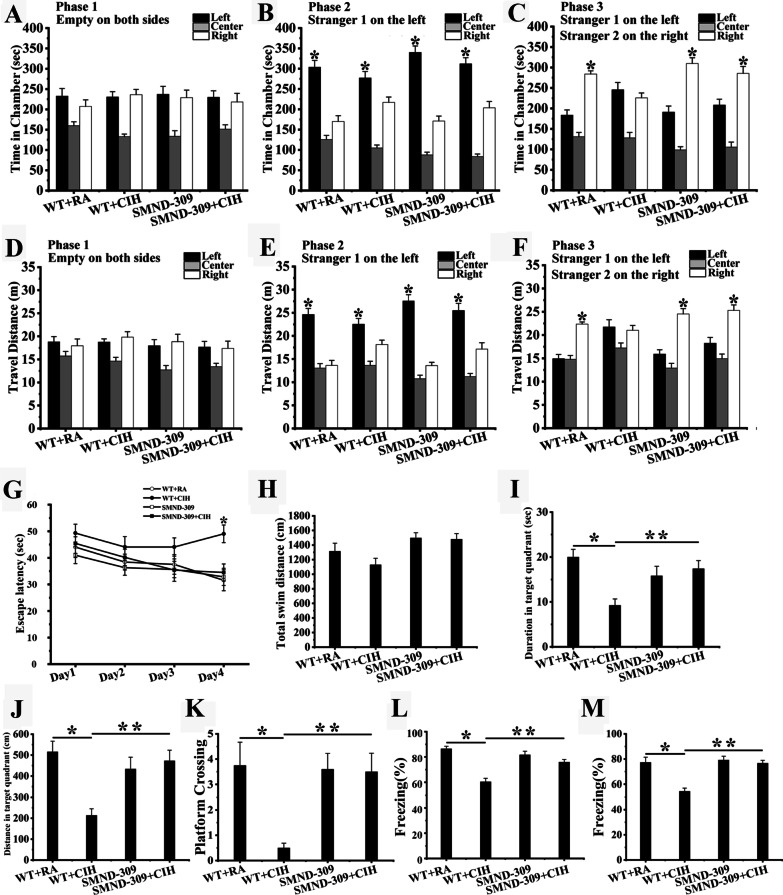
The behavioral performance in mice. **A**, **D** During phase 1, there was no significant difference between the two sides in all groups. **B**, **E** During phase 2, all of the mice preferred to stay with the stranger mouse. **C**, **F** During phase 3, WT + RA, SMND-309 and SMND-309 + CIH mice preferred to stay with Stranger 2 than with Stranger 1 (p < 0.05). However, WT + CIH mice spent a similar amount of time with each of the two stranger mice (p > 0.05). **G** The mean escape latencies of the four groups. **H** The total swimming distances among all groups were similar (p > 0.05). **I** The time the WT + CIH mice spent in the target quadrant was much shorter than the time the WT + RA mice spent (p < 0.05), and this was improved by SMND-309 treatment (p < 0.05). **J** WT + RA mice traveled longer distances in the target quadrant than the WT + CIH mice (p < 0.05), and after SMND-309 treatment, the damage was improved (p < 0.05). **K** The number of platform crossings of WT + CIH mice was less than WT + RA mice (p < 0.05), but it was improved by treating with SMND-309 (p < 0.05). **L**, **M** The fear memory was damaged by CIH exposure, and SMND-309 alleviated the damage. *p < 0.05 Stranger 1 vs. empty cage. *p < 0.05 Stranger 1 vs. Stranger 2. *p < 0.05 WT group vs. WT CIH group. **p < 0.05 WT CIH group vs. SMND-309 CIH group. N = 10 for each group. Data are shown as the mean ± SEM. The results of social tests were analyzed by one-way ANOVA and a Bonferroni test. The results of escape latency were checked by three-way mixed ANOVA. Others were measured by two-way ANOVA

At phase 1, there was no obvious side preference in the 4 groups (shown in Fig. 2A, D). During phase 2, all the mice preferred to stay with the stranger mouse than at the inanimate side (p < 0.05, shown in Fig. 2B, E). At phase 3, WT + RA mice (F_(2, 27)_ = 55.839, T = 6.827, p < 0.05) and SMND-309 mice (F_(2, 27)_ = 71.527, T = 6717, p < 0.05) preferred to stay with the new stranger mouse than the familiar one (shown in Fig. 2C). However, WT + CIH mice spent similar time at the two sides (F_(2, 27)_ = 18.716, p > 0.05) (shown in Fig. 2C) during this stage. Treated with SMND-309, SMND-309 + CIH mice spend increasing time with the stranger mouse than the familiar one (F_(2, 27)_ = 37.689, T = 3.718, p < 0.05) (shown in Fig. 2C). The traveling distance of phase 3 showed same pattern with spending time for all groups (shown in Fig. 2F). These results showed that CIH treatment damaged the social novelty cognition of mice and SMND-309 could alleviate the damage.

### CIH exposure induced spatial learning and memory deficits, SMND-309 could relieve

During the training days, the mean escape latency of WT + RA and SMND-309 mice decreased rapidly, but it had almost no change in the WT + CIH group (day 4: F_(3,156)_ = 5.626, T = 3.585, p < 0.05) (shown in Fig. 2G). At the probe trial stage, the total swimming distance of all groups was similar (F_(3, 36)_ = 1.564, p = 0.215) (shown in Fig. 2H). In contrast, the WT + CIH group showed a remarkable reduction in the number of platform crossings (F_(3, 36)_ = 3.28, T = 3.725, p < 0.05) (shown in Fig. 2I). The duration (F_(3, 36)_ = 7.266, T = 3.939, p < 0.05) (shown in Fig. 2J) and the distance (F_(3, 36)_ = 4.007, T = 3.963, p < 0.05) (shown in Fig. 2K) traveled at the target quadrant were also shorter for the WT + CIH group than for the WT + RA group. After SMND-309 treatment, these reductions were notably reversed. These data indicated that CIH exposure impair the spatial learning and memory of mice, but SMND-309 relieved the damage.

### CIH exposure induced fear memory impairment, SMND-309 could improve

Twenty-four hours after the footshock, WT + CIH mice presented with a lower level of freezing than WT + RA mice (F_(3, 36)_ = 14.139, T = 6.82, p < 0.05), but this change was improved after treatment with SMND-309 in SMND-309 + CIH group (F_(3, 36)_ = 14.139, T = 4.079, p < 0.05) (shown in Fig. 2L). Similar patterns could also be observed in the cued memory test (day 3) (shown in Fig. 2M). In conclusion, the fear memory of mice was severely injured under CIH condition, and this injure could be ameliorated by SMND-309 treatment.

### CIH-induced pathological changes in the hippocampus

**Fig. 3:**
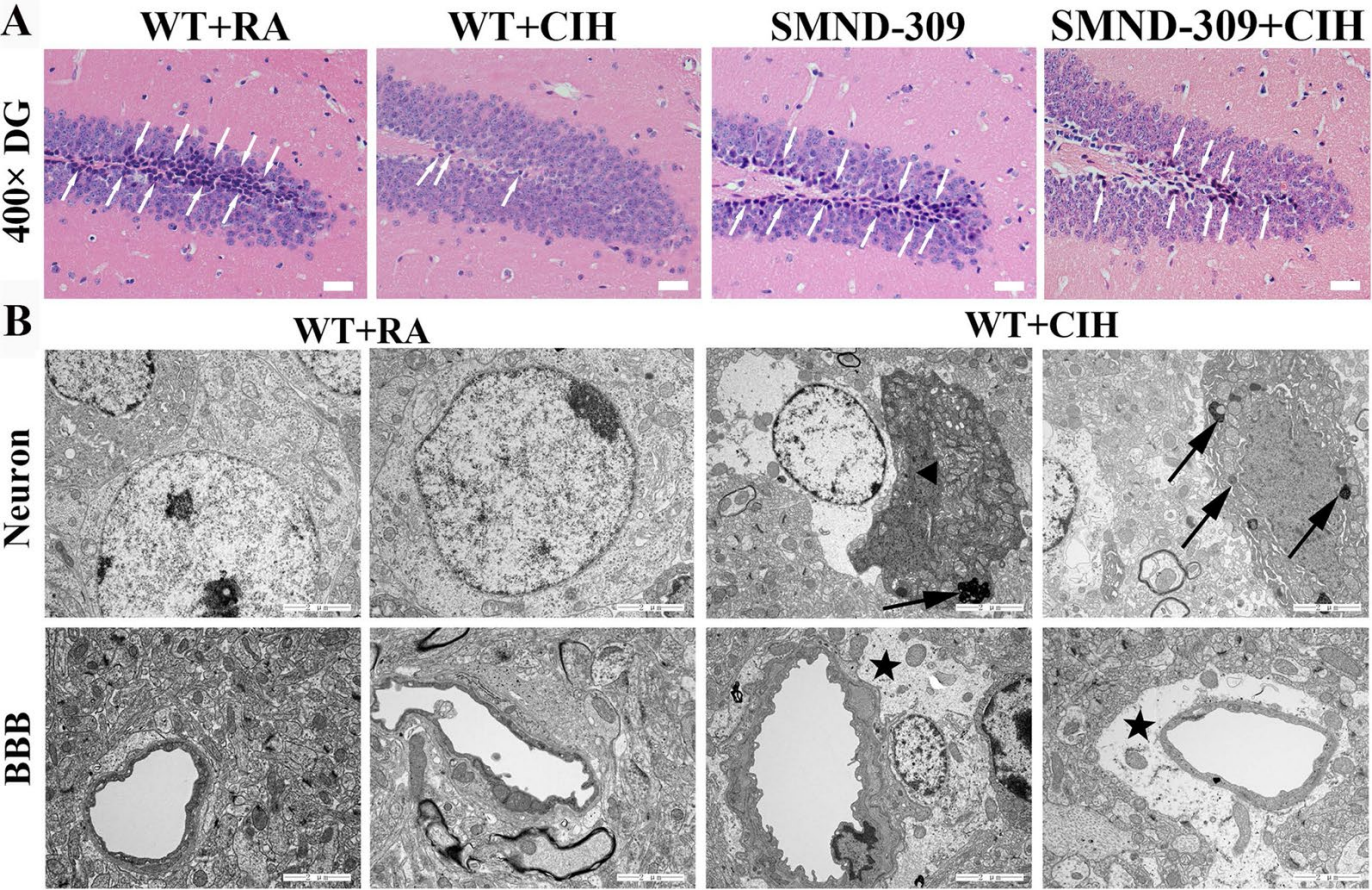
Pathological changes of DG area. **A** H&E staining of the hippocampus. In the SGZ of the WT + CIH group, NBs were hardly observed (White arrow), but SMND-309 treatment relieved the damage. Original magnification: 400×. **B** The TEM results of the hippocampus. Neurons of the WT + CIH group exhibited cytoplasmic vacuolization, LDs accumulation (black arrow) and mitochondrial disruption (black triangle). Moreover, glial cells of the WT + CIH group presented edema (black star), especially in the area surrounding the BBB. Original magnification: 15,000×. All experiments were repeated three times

After CIH treatment, severe histological changes have been revealed, neurons are loosely arranged, irregular sized, with fuzzy outline. Meanwhile, the number of neuroblasts (NBs) in the hippocampal subgranular zone (SGZ) was significantly decreased (shown in Fig. 3A). This injury was relieved by SMND-309 treatment (shown in Fig. 3A). As seen with TEM, the neuron ultrastructure was completed, and the blood–brain barrier (BBB) was thin and intact in the WT hippocampus (shown in Fig. 3B). However, neurons exhibited cytoplasmic vacuolization and mitochondrial disintegration under CIH conditions. Moreover, a large number of LDs appeared in the damaged neurons (shown in Fig. 3B). For astrocytes, severe edema could be observed, especially in the area surrounding the BBB (shown in Fig. 3B).

### LDs-induced NBs apoptosis after CIH exposure, SMND-309 could ameliorate

**Fig. 4:**
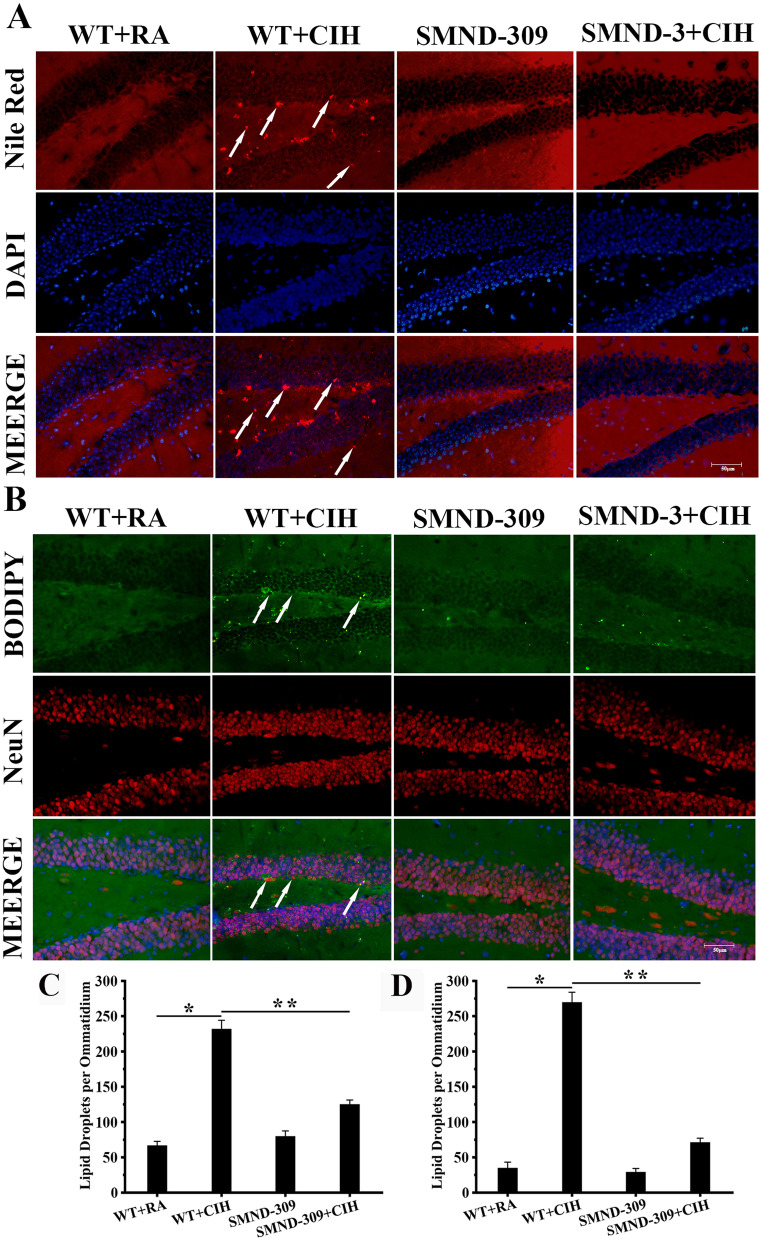
LDs accumulation in neuron. To detect LDs accumulation in the hippocampal neurons, Nile red (red) and DAPI (blue), BODIPY (483/503) (green) and NeuN (red) were used. **A** LDs accumulation could be detected (red, black arrow) in the WT + CIH mice, and treated with SMND-309 decreased this change. **B** Abundant BODIPY (483/503) fluorescence could be detected in the neurons of the DG area under CIH condition, but the fluorescence became dim after SMND-309 treatment. **C** Quantification of LDs accumulation in nerve and glia cells. **D** Quantification of LDs accumulation in neurons. *p < 0.05 WT group vs. WT CIH group. **p < 0.05 WT CIH group vs. SMND-309 CIH group. All experiments were repeated three times. Data are shown as the mean ± SEM. Statistical analysis was tested by two-way ANOVA. Original magnification: 400×

**Fig. 5:**
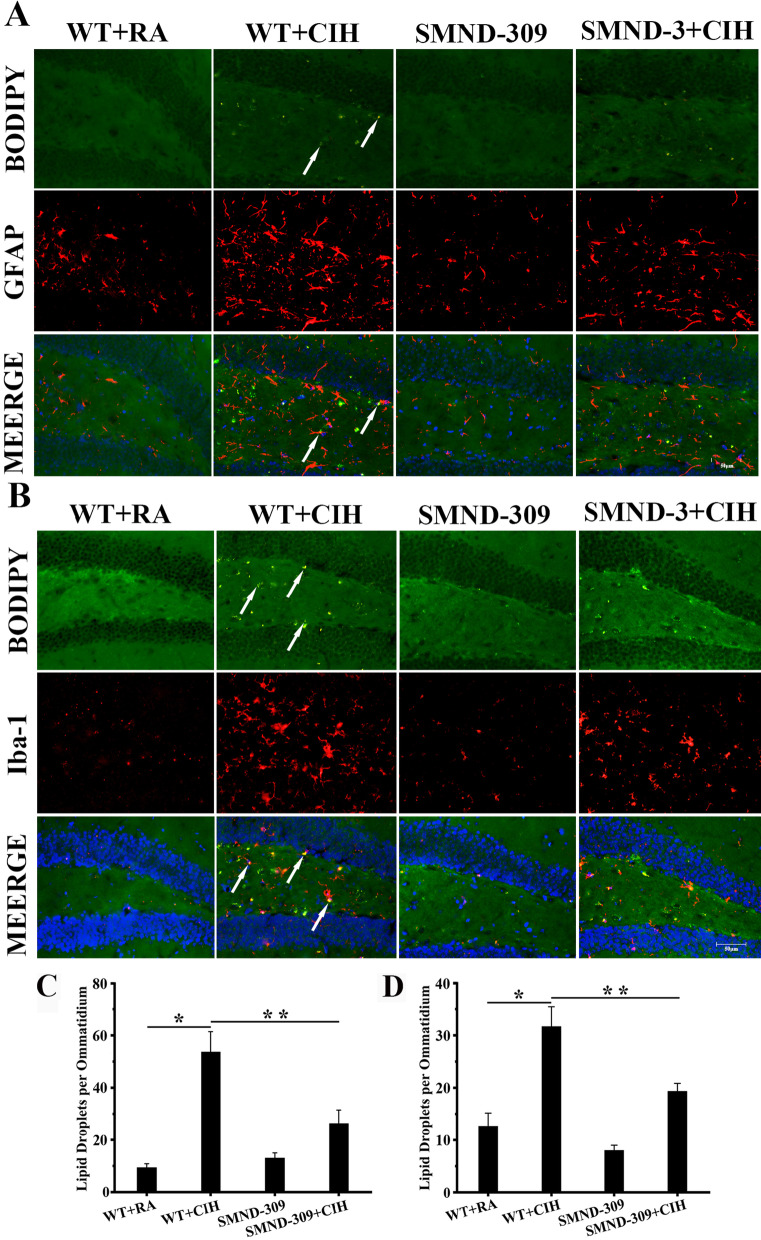
LDs accumulation in glial cells. The microglia marker Iba-1 (red), astrocyte marker GFAP (red) and DAPI (blue) were stained with BODIPY (483/503) (green) to observe LDs accumulation in glia. The swollen cell body and increased synapses along with the mass of BODIPY (483/503) spots could be observed in glial cells after CIH exposure. With SMND-309 treatment, glial injuries were relieved. LDs accumulated (yellow, black arrow) could also be detected in astrocytes (**A**) and microglia (**B**). **C** Quantification of LDs accumulation in astrocytes. **D** Quantification of LDs accumulation in microglia. *p < 0.05 WT group vs. WT CIH group. **p < 0.05 WT CIH group vs. SMND-309 CIH group. All experiments were repeated three times. Data are shown as the mean ± SEM. Statistical analysis included two-way ANOVA. Original magnification: 400×

The fluorescence of Nile red was dim in the dentate gyrus (DG) area of WT + RA mice and SMND-309 mice (shown in Fig. 4A), but the number of LDs increased twofold after CIH treatment (p < 0.05). Interestingly, it decreased 45% after SMND-309 treating in SMND-309 + CIH group (p < 0.05) (shown in Fig. 4A). To confirm the distribution of LDs in different types of nerve and glia cells, cell-type markers NeuN, GFAP, Iba-1 and LDs dye BODIPY (483/503) were used. Following CIH treatment, the fluorescence spots of BODIPY (483/503) enhanced eightfold in the neurons (p < 0.05) (shown in Fig. 4B), nearly threefold in the astrocytes (p < 0.05) (shown in Fig. 5) and onefold in the microglia (p < 0.05) (shown in Fig. 5) of the DG area than WT + RA group. Intriguingly, SMND-309 noticeably decreased 70% of LDs accumulation in the neurons (p < 0.05) (shown in Fig. 4B), 40% in the astrocytes (p < 0.05) (shown in Fig. 5) and 30% in the microglia (p < 0.05) (shown in Fig. 5).

**Fig. 6:**
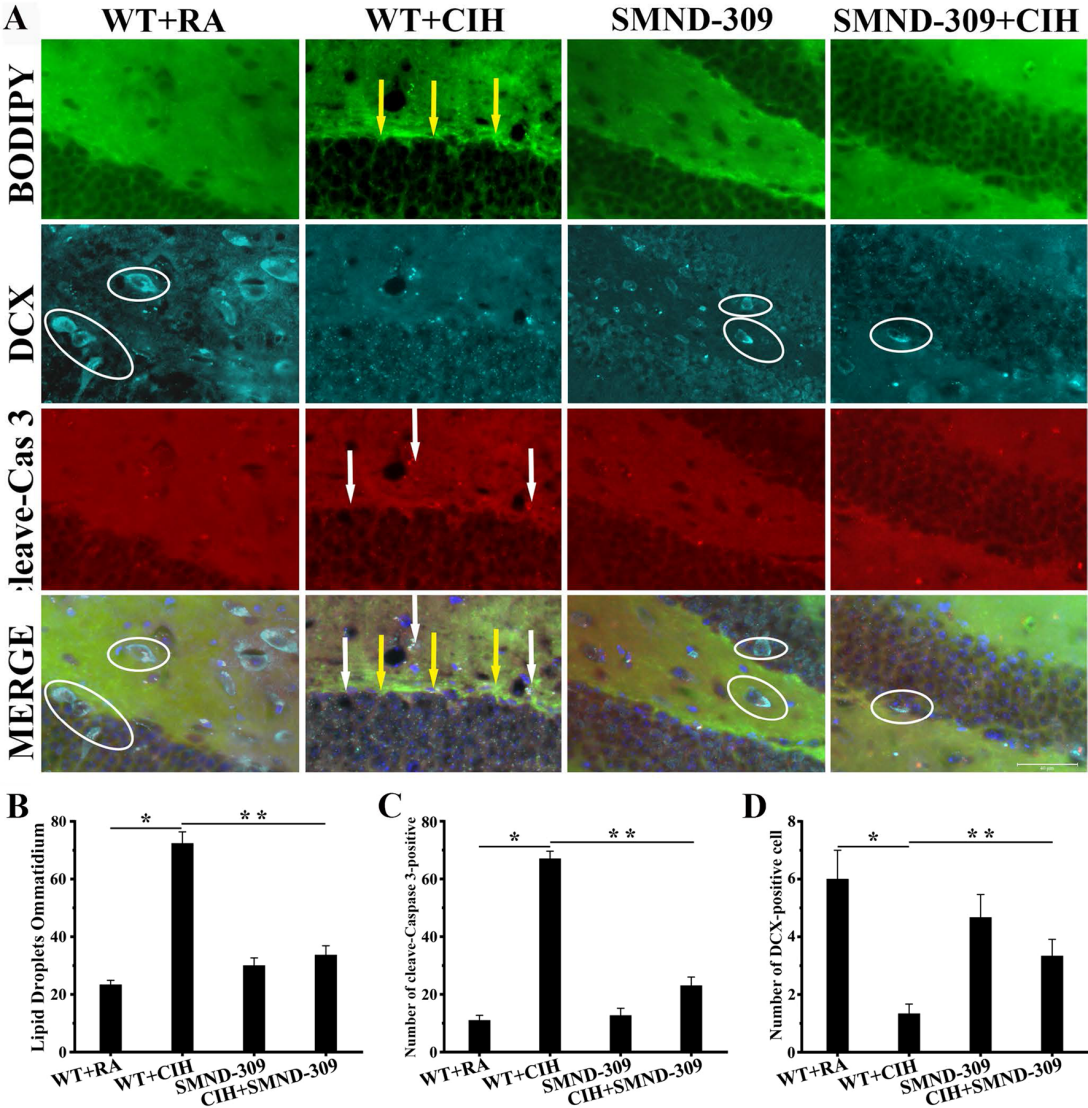
LDs accumulation-induced NBs apoptosis. Fluorescent immunocytochemistry staining was as follows: NB marker DCX (sky blue), cleaved caspase 3 (red), BODIPY (483/503) (green) and DAPI (blue). **A** Seldom DCX-positive cells (blue, white circle) in the SGZ of WT + CIH mice could be observed. Moreover, a larger number of BODIPY (483/503) (green, yellow arrow) and cleaved caspase 3-positive cells (red, white arrow) merged. SMND-309 treatment ameliorated these injuries. **B** Quantification of LDs accumulation. **C** Quantification of cleaved caspase 3-positive cell. **D** Quantification of DCX-positive cell. *p < 0.05 WT group vs. WT CIH group. **p < 0.05 WT CIH group vs. SMND-309 CIH group. All experiments were repeated three times. Data are shown as the mean ± SEM. Statistical analysis was performed with two-way ANOVA. Original magnification: 600×

To determine the damage of NBs in the SGZ by LDs accumulation, the NB marker DCX and the apoptosis marker cleave-caspase 3 was stained with the LDs dye BODIPY (483/503). The number of DCX-positive cells in the DG area was decreased 80% after CIH treatment (p < 0.05) (shown in Fig. 6). In addition, the quantity for cleaved caspase 3-positive cell increased 85% with BODIPY (483/503) spots in the DG area of WT + CIH mice (p < 0.05) (shown in Fig. 6). Notably, apoptosis NB significantly reduced in SMND-309 + CIH group than WT + CIH group (p < 0.05) (shown in Fig. 6).

### Affecting the pathway of PDP1/PDHA1 did not improve the damage caused by CIH exposure

**Fig. 7:**
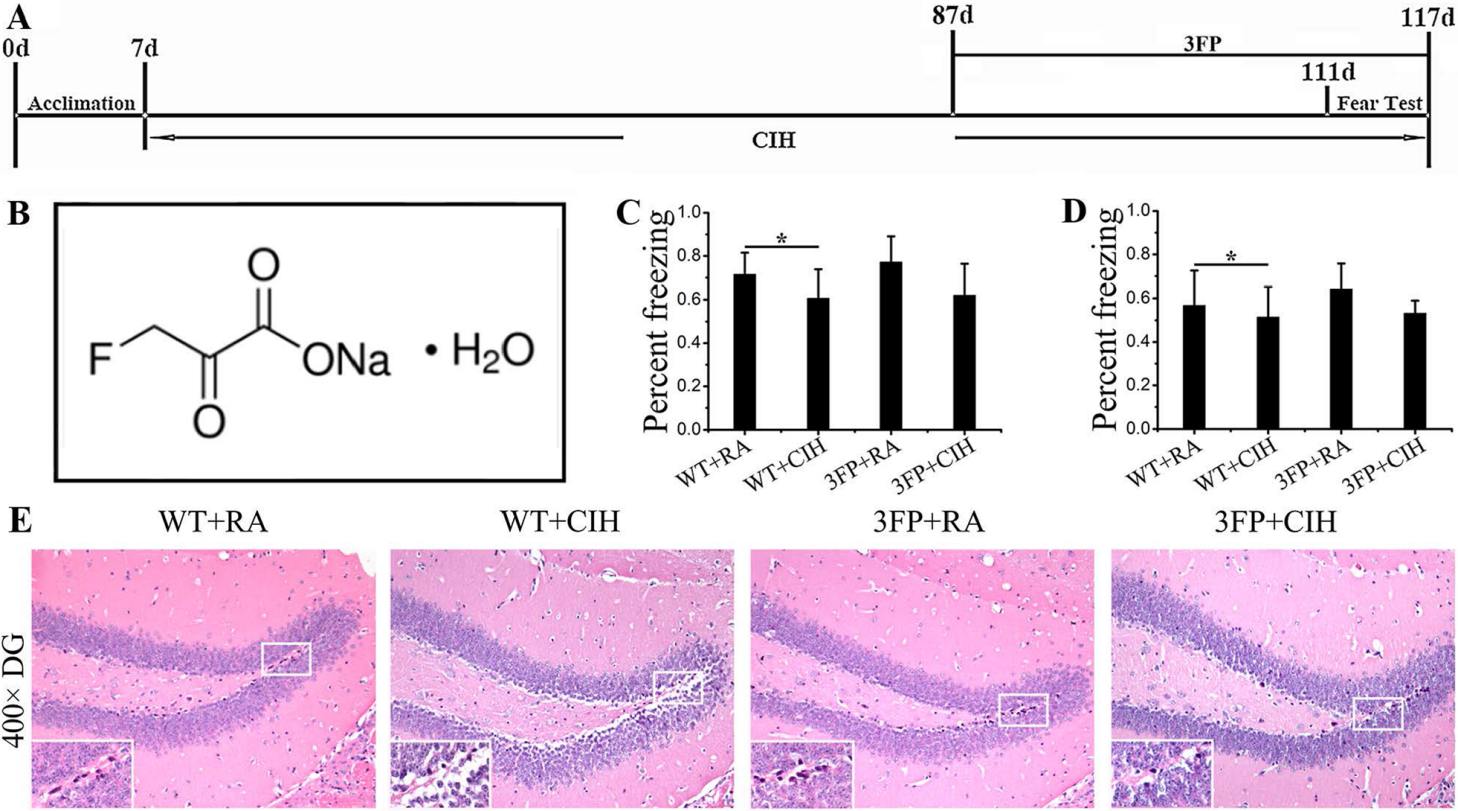
Affecting the pathway of PDP1/PDHA1 did not improve the damage caused by CIH exposure. **A** The study workflow. After 7 days of acclimation, the mice were exposed to the CIH condition for about 3 months, and they were treated with 3-FP at the last 3 weeks. Fear test were conducted as soon as CIH exposure finished. **B** The structure of 3-FP. **C** The freezing level of contextual memory among 4 groups. **D** The freezing level of cued memory among 4 groups. **E** H&E stained of the hippocampus. Each experiment was repeated three times. Data are shown as the mean ± SEM. Statistical analysis was performed with two-way ANOVA. *p < 0.05 WT group vs WT CIH group. Magnification: 400×. The local details are enlarged in the rectangular frame

The mice were exposed to RA/CIH condition for about 3 months, and received intraperitoneal injection with 3-FP every 3 days at the last 3 weeks of the exposure(shown in Fig. 7A). The fear conditioning test were conducted as soon as the treatment was finished (shown in Fig. 7A).

In the context memory test, WT + CIH mice presented with a lower level of freezing compared with WT + RA group than mice. It should be noted that this change was not improved after treatment with PDHA1 inhibitor 3-FP (shown in Fig. 7C, *p < 0.05). After 48 h retention delay, the cued fear conditioning testing were determined, the freezing level was similar to the trend of context memory test (shown in Fig. 7D, *p < 0.05). Taken together, these results provided evidence that CIH exposure contributed to the long-term memory dysfunction, and PDHA1 inhibitor 3-FP could not improve the damage.

The H&E stained of hippocampus section in 4 groups were shown in Fig. 7E. Compared to the RA group, severe histological changes had been revealed in CIH group. Hippocampal granular neurons were loosely arranged, some neurons had showed a swollen and vacuolated cytoplasm and the neural progenitor cells in the DG area were significantly reduced. Significantly, this injury was not relieved by PDHA1 inhibitor treatment.

### PDP1/PDHA1 did not significantly promote the increase of LDs induced by CIH in hippocampus

**Fig. 8:**
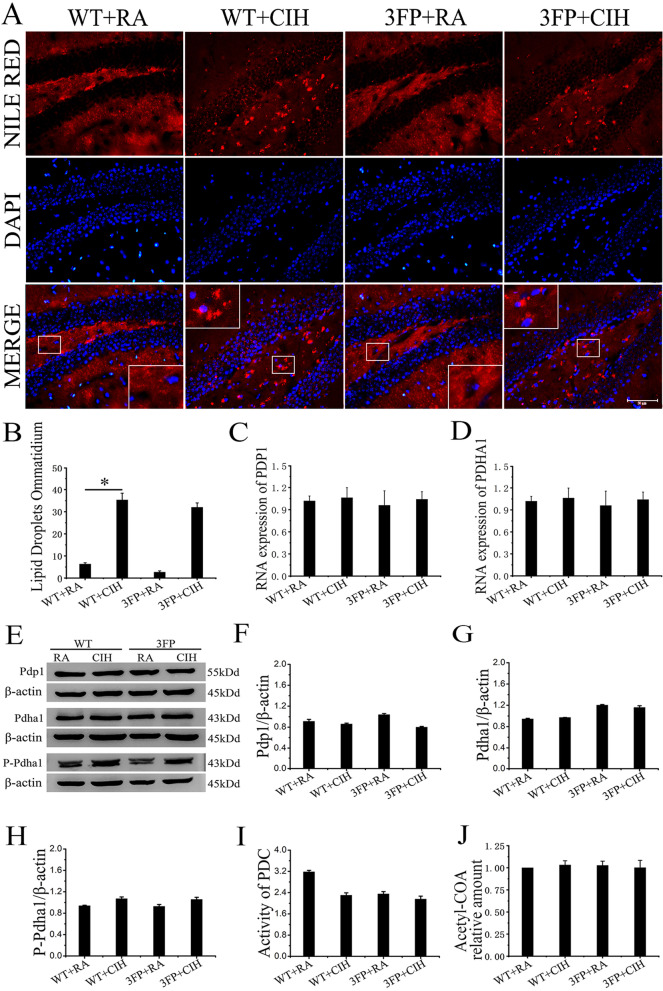
CIH treatment did not significant influence the expression of PDP1/PDHA1 in hippocampus. **A** Nile Red staining of hippocampus. (Scale bar: 20 μm). **B** Quantification of LDs accumulation. **C**, **D** PDP1 and PDHA1 expression patterns were determined by RT-PCR. **E**–**H** Western blot assays were performed to determine the expression level of PDP1/PDHA1, and the gray value of the band was measured. **I** The PDC activity of hippocampus. **J** Total levels of acetyl-CoA in hippocampus. Each experiment was repeated three times. Data are shown as the mean ± SEM. Statistical analysis was performed with two-way ANOVA. *p < 0.05 WT group vs WT CIH group. Magnification: 400×. The local details are enlarged in the rectangular frame

The fluorescence of Nile red showed that the CIH group and CIH + 3-FP group had sporadic superficial, round or elongated lipid-containing structures in the hippocampus, indicating abnormal accumulation of LDs (shown in Fig. 8A, B). Real time RT-PCR analysis and Western blot analysis were performed to analyze the expression level of PDP1 and PDHA1. Quantitatively, the gene expression and protein levels of PDP1 and PDHA1 showed no significant differences among 4 groups (shown in Fig. 8C–G). The protein level of phosphorylated PDHA1 (p-PDHA1) (shown in Fig. 8H), the activity of PDC (shown in Fig. 8I) and the content of Acetyl-CoA (shown in Fig. 8J) were tested to further explore the role of PDP1/PDHA1 related lipid synthesis on CIH condition. All these results showed no statistically differences between groups, which suggested CIH exposure almost had no impact on the Acetyl-CoA biosynthesis regulated by PDP1/PDHA1. In vitro experiments supported this conclusion (shown in Additional file 1).

Experimental data showed that the PDP1/PDHA1 may not be the main source of the abnormal increased lipids after CIH exposure. Therefore, other reasonable mechanisms of lipids synthesis disorder in nerve and glia cells under stress needed to be interpreted.

### ROS-triggered JNK/SREBP/ACC pathway activation and lipid peroxidation, SMND-309 could decrease

**Fig. 9:**
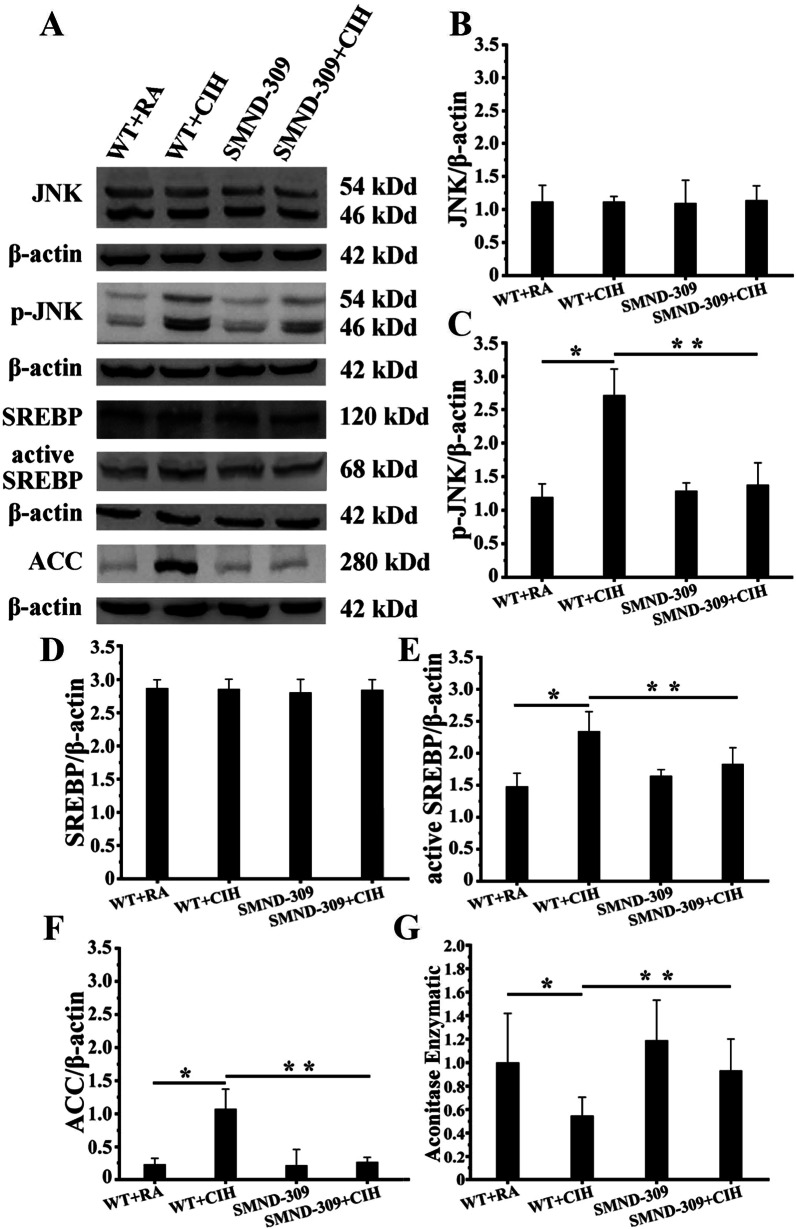
ROS level and JNK/SREBP/ACC expression. **A**–**G** The ROS level was highly enhanced in the hippocampus of WT + CIH exposure (p < 0.05), and the JNK/SREBP/ACC pathway was activated. However, these changes were attenuated by SMND-309 treatment (p < 0.05). *p < 0.05 WT group vs. WT CIH group. **p < 0.05 WT CIH group vs. SMND-309 CIH group. All experiments were repeated three times. Data are expressed as the mean ± SEM. Statistical analysis was performed through two-way ANOVA

To confirm the level of ROS in the hippocampus under CIH conditions, an aconitase activity assay kit was used. The results showed that the activity for aconitase decreased 50% in WT + CIH group (p < 0.05) (shown in Fig. 9G). Furthermore, the expression of phosphorylated JNK increased nearly 1.5-fold (p < 0.05), and this effect activated SREBP and upregulated the expression of ACC for threefold (p < 0.05) (shown in Fig. 9A–F). Treated with SMND-309 eliminated ROS (p < 0.05) (shown in Fig. 9G) and inhibited the activation of the JNK/SREBP/ACC pathway (shown in Fig. 9A–F).

**Fig. 10:**
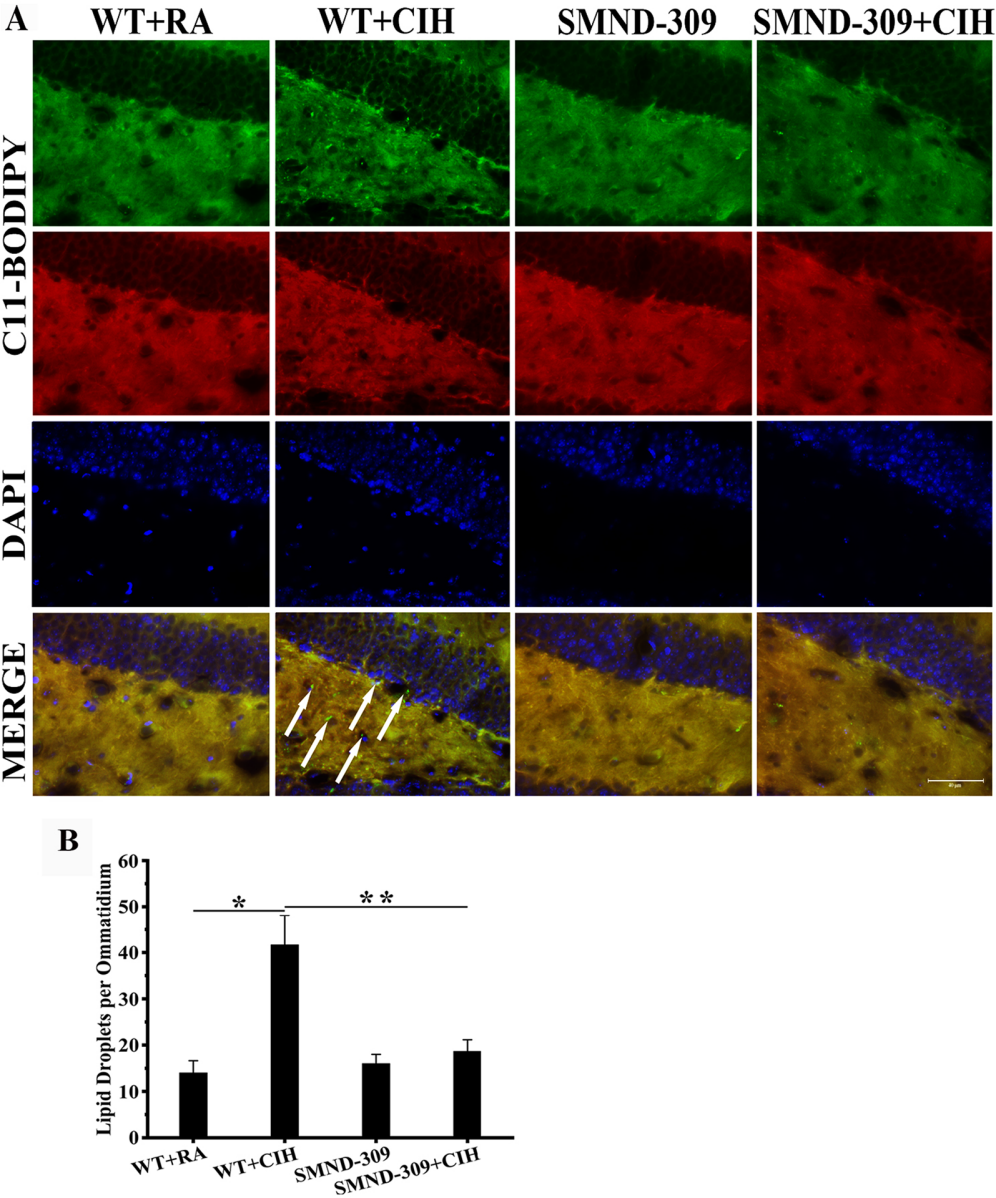
Lipid peroxidation in nerve and glia cells. **A** C11-BODIPY (581/591) (red, green) and DAPI (blue) staining of the DG region. Lipid peroxidation could be observed (green, white arrow) in nerve and glia cells and SMND-309 inhibited peroxidation. **B** Quantification of peroxidized lipid in the nerve and glia cells. *p < 0.05 WT group vs. WT CIH group. **p < 0.05 WT CIH group vs. SMND-309 CIH group. All experiments were repeated three times. Data are shown as the mean ± SEM. Statistical analysis was performed with two-way ANOVA. Original magnification: 400×

ROS are oxidation products and cause damage to lipids. Peroxidized lipids could be observed after staining with the lipid peroxidation dye C11-BODIOY (581/591), results indicated that the number of peroxidated lipid increased twofold in the hippocampal nerve and glia cells of WT + CIH mice (p < 0.05) (shown in Fig. 10). Notably, it reduced nearly 50% after SMND-309 treatment (p < 0.05).

## Discussion

As a common sleep disorder disease, OSAS is characterized by sudden pauses of breathing during sleep (Khokhrina et al. 2020). Resulting from repeated obstructions of the pharyngeal airway, CIH is a cardinal feature of OSAS, which induces degrease of cognitive performance (Li et al. 2020). Our research found that the ability of social memory and spatial memory was damaged in C57BL/6 mouse after CIH treatment. Further study discovered that the hippocampal nerve and glia cells of those mice were severely injured. It is thought that hippocampus is crucial for encoding new memory (Cascella and Al Khalili 2021). Therefore, the injury of hippocampus by CIH finally gave rise to behavioral deficits. Until now, researchers consider that the mechanism of those neuro damage is related to the inflammation and the oxidative stress. In response to CIH, immune receptors initiate chronic neuroinflammation, such as TLR 2 and TLR 4. Those receptors upregulate the expression of inflammation cytokines, including IL-1β, IL-6 and TNF-α via TLR 2/TLR 4-MyD88 signal pathway (Li et al. 2020) (Lu et al. 2019). In this process, microRNA (Ren et al. 2019), histone modifications and DNA methylation (Kiernan et al. 2016) have also been involved in this process. Besides, hypoxia-reoxygenation damage cellular organelles and structures by promoting ROS generation. Targeting mitochondrial protein OPA1, ROS induces mitochondrial fission and disturbs mitochondrial membrane potential (Rovira-Llopis et al. 2017). Also, ROS initials apoptosis by promoting ER calcium release and triggering ER stress (Ding et al. 2016).

Recently, LDs accumulation is closely related to a variety of human diseases (Onal et al. 2017). In peripheral system, cell death-inducing DFF45-like effector (CIDE), which is crucial for the formation and fusion of LDs, regulates the occurrence of type II diabetes (Zhang et al. 2011). The depletion of perilipin 2 prevents hepatic steatosis via downregulating triglyceride synthesis and LDs accumulation (Carr et al. 2014). In nervous system, LDs accumulation enhances the formation of oligomeric α-synuclein, a major component of the pathological hallmarks in Parkinson's disease (Ruipérez and Darios 2010). LDs accumulation also disrupts energy homeostasis (Konige et al. 2014), impairs the folding and clearance of proteins in neurons (Inloes et al. 2018), and breaks neuron-glia metabolic coupling (Schmitt et al. 2014). All these studies have shown that LDs accumulation is closely related to neurodegeneration diseases. Contradictorily, some studies support that LDs accumulation is helpful for neural protection. In the glial niche of *Drosophila* larvae, LDs accumulation keeps neural stem cells away from oxidative damage (Moldavski et al. 2015). Some fatty acids are vulnerable to peroxidation, and they are diverted into LDs to protect from ROS under hypoxia condition (Welte and Gould 2017). Therefore, exploring the molecular mechanisms of altered lipid metabolism in brain injury, will help to reveal the cause of neurodegenerative changes, including CIH-induced cognitive dysfunction.

PDP1/PDHA1 pathway is an essential regulatory for de novo lipid synthesis. PDHA1 is activated by dephosphorylation of PDP1 and phosphorylation of pyruvate dehydrogenase kinases (PDK) (Shan et al. 2014). PDHA1 is one of the most important components of the PDC (Zhong et al. 2017), which oxidates pyruvate to Acetyl-CoA. As a major and central precursor in metabolism, Acetyl-CoA candidates in the synthesis and decomposition of biomacromolecules, especially for lipid biosynthesis (Kuerschner et al. 2008). It has been reported that PDP1/PDHA1 was related to a variety of diseases by affecting lipid metabolism. Inactivation of PDHA1 suppresses tumourigenesis by decreasing Acetyl-CoA levels in prostate cancer (Chen et al. 2018). However, knocking out PDK4 alleviates the hepatic steatosis by regulating the activity of PDC in nonalcoholic steatohepatitis mouse models (Zhang et al. 2018). In this study, experimental data showed that the activity of PDC and the production of Acetyl-CoA did not noticeable change after CIH exposure, which suggested the de novo lipid synthesis regulated by PDP1/PDHA1 might not be the main source of the abnormal increased lipids and LDs after CIH exposure. Therefore, the relevant mechanism still needs to be further studied.

ROS production and oxidative stress participate in neuro injuries and neurodegeneration. In Parkinson disease, ROS induces missense mutation by damaging DNA and causing neural cell damage (Pignataro et al. 2017). ROS also evokes Alzheimer's disease through active NLRP3, which promotes IL-1β-mediated inflammation (Pignataro et al. 2017). Recently, studies found that ROS is capable to promote lipid synthesis and LDs accumulation (Liu et al. 2015). Increased level of ROS exerts harmful effects by causing oxidative damage to biological macromolecules and disrupting various signaling pathways including the lipid metabolism (Fransen et al. 2012). In the development of fatty liver, redox cellular state especially the high level of ROS activates lipid biosynthesis gene SREBP and speeds up the disease process (Pan et al. 2017). Reports show that ROS triggers SREBP activity in fruit fly neurons and leads to LDs accumulation (Liu et al. 2015). SREBP regulates the expression of several genes, such as ACC and fatty acid synthase (FAS) (Yuan et al. 2019). ACC is a rate-limiting enzyme in de novo fatty acid synthesis, catalyzing ATP-dependent carboxylation of Acetyl-CoA to form malonyl-CoA (an intermediate in fatty acid biosynthesis) (Hunkeler et al. 2018). We found that high level of ROS triggered JNK/SREBP/ACC pathway in neurons after CIH exposure. The excessive increase of lipid synthesis promoted abnormal LDs accumulation, which severely injured nerve and glia cells in hippocampus. In addition, lipid peroxidation further aggravated neuro damage.

SMND-309 is a degradation production of *Salvia miltiorrhiza*, which has been used for neuroprotection (Su et al. 2015). SMND-309 inhibits apoptosis by upregulating the ratio of Bcl-2/Bax (Yang et al. 2010) and promotes neuron survival by increasing the content of brain-derived neurotrophic factor via activating the phosphatidylinositol 3-kinase/Akt/cAMP-response element-binding (CREB) signaling pathway (Wang et al. 2016). Consistent with the results of previous studies, SMND-309 treatment improved the behavioral performance of CIH mice by reducing the accumulation of LDs in nerve and glia cells of the DG area. These findings might be helpful to provide a novel potential neuroprotective therapy.

In this study, CIH-induced hippocampal damage was triggered by LDs accumulation in NBs, neurons and glia cells. The generation of LDs could be regulated via JNK/SREBP/ACC pathway. And these damages were alleviated by SMND-309 treatment. Until now, the role of LDs in neurocyte is controversial. The mechanism of lipid synthesis disorder and LDs abnormal accumulation is remain unclear. All these questions need to be further investigated.

## Conclusion

Present study found that cognitive function of CIH wice was severely damaged because of neurocyte injuries in the hippocampal. After CIH exposure, the expression of PDP1/PDHA1, the activity of PDC and the level of cellular Acetyl-CoA have barely changed. Noticeably, ROS triggered JNK/SREBP/ACC pathway and led to aberrant LDs accumulation, which contributed to the neuro injuries. What’s more, lipid peroxidation as a result of excessive ROS also aggravated the damage in nerve and glia cells. Noticeably, the neural damages induced by lipid metabolic disorders were relieved by SMND-309 treatment.

## Supplementary Information

**Additional file 1: S1**. CIH treatment did not significant influence the expression of PDP1/PDHA1 in *vitro.* (A, B) The IH program and the sequence of experiment process. (C) BODIPY (green) and DAPI (blue) staining of the HT22. Magnification:600×. (D-F) The number of LDs per cell. Compared with the NC group, the number of LDs in the IH group were obviously increased. However, treated with 3-FP could not reduce the number of LDs. (G, H) Quantitative real-time PCR experiments showed the expression levels of PDP1 and PDHA1 in Ht22 cells. The results showed no statistically difference among four groups. (I-L) The PDP1/PDHA1 pathway was activated by Western blot, and no statistically difference among four groups. (M) The activity of PDC and no significant difference could be found. All experiments were repeated three times. Data are expressed as the mean ± SEM. Statistical analysis was performed through two-way ANOVA. The treated samples were different from the controls at p < 0.05.

## Data Availability

The data that support the findings of this study are available from the corresponding author upon reasonable request.

## Abbreviations

CIH: Chronic intermittent hypoxia
IH: Intermittent hypoxia
LDs: Lipid droplets
NBs: Neuroblasts
JNK: C-Jun-N-terminal Kinase
SREBP: Sterol regulatory element binding protein
ACC: Acetyl CoA carboxylase
OSAS: Obstructive sleep apnea syndrome
SGZ: Subgranular zone
HSP: Hereditary spastic paraplegia
PDP1: Pyruvate dehydrogenase phosphatase 1
PDHA1: Pyruvate dehydrogenase complex E1ɑ subunit
PDC: Pyruvate dehydrogenase complex
GFAP: Glial fibrillary acidic protein
DCX: Doublecortin
WT: Wild type
RA: Room air
NC: Normal control
3-FP: 3-Fluoropyruvate
MWM: Morris water maze
TEM: Transmission electron microscopy
H&E: Hematoxylin and eosin
ANOVA: Analysis of variance
BBB: Blood–brain barrier
CIDE: Cell death-inducing DFF45-like effector
PDK: Pyruvate dehydrogenase kinases
PSA-NCAM: Polysialylated form of the neural cell adhesion molecule
CREB: CAMP-response element-binding
