# Supplementary material for: Abnormal lipid droplets accumulation induced cognitive deficits in obstructive sleep apnea syndrome mice via JNK/SREBP/ACC pathway but not through PDP1/PDC pathway
Source: Mol Med. 2022 Jan 14;28:3. doi: 10.1186/s10020-021-00427-8 (PMC8760803; doi:10.1186/s10020-021-00427-8)
Supplement: Supplementary file 1 — Additional file 1: S1. CIH treatment did not significant influence the expression of PDP1/PDHA1 in vitro. (A, B) The IH program and the sequence of experiment process. (C) BODIPY (green) and DAPI (blue) staining of the HT22. Magnification:600×. (D-F) The number of LDs per cell. Compared with the NC group, the number of LDs in the IH group were obviously increased. However, treated with 3-FP could not reduce the number of LDs. (G, H) Quantitative real-time PCR experiments showed the expression levels of PDP1 and PDHA1 in Ht22 cells. The results showed no statistically difference among four groups. (I-L) The PDP1/PDHA1 pathway was activated by Western blot, and no statistically difference among four groups. (M) The activity of PDC and no significant difference could be found. All experiments were repeated three times. Data are expressed as the mean ± SEM. Statistical analysis was performed through two-way ANOVA. The treated samples were different from the controls at p < 0.05. [file 10020_2021_427_MOESM1_ESM.docx]

**Supplement**

**Abnormal lipid droplets accumulation induced cognitive deficits in obstructive sleep apnea syndrome mice via JNK/SREBP/ACC pathway but not through PDP1/PDC pathway**

Dongze Li^1*^, Na Xu^1*^, Yanyan Hou^1^, Wenjing Ren^1^, Na Zhang^1^, Xi Wang^2^, Yeying Sun^1^, Wenxue Lu^1^, Guiwu Qu^1^, Yan Yu^1*#^, Changjun Lv^1#^, Fang Han^1#^


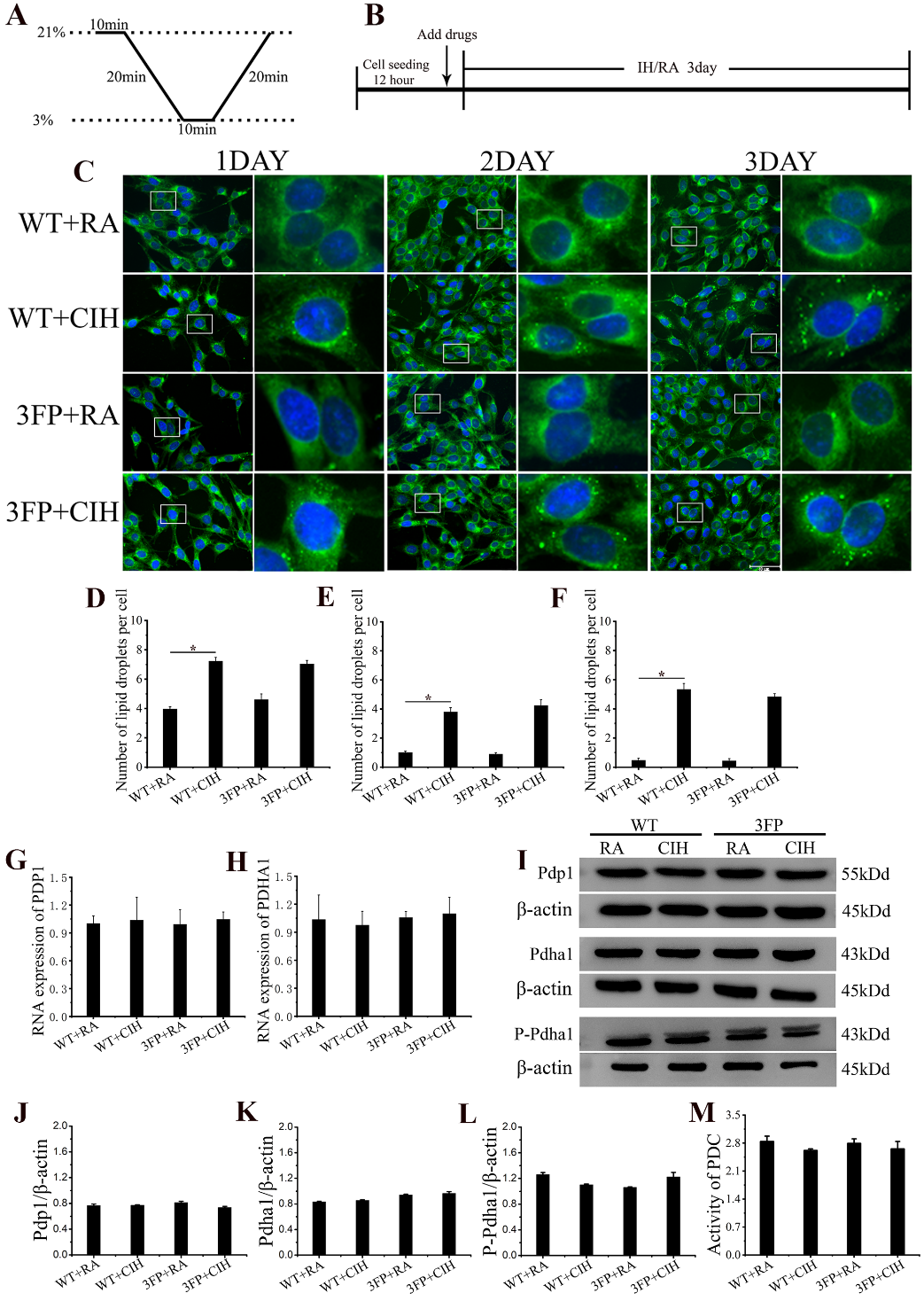


**Supplement. 1.** **CIH treatment did not significant influence the expression of PDP1/PDHA1 in *vitro.*** (A, B) The IH program and the sequence of experiment process. (C) BODIPY (green) and DAPI (blue) staining of the HT22. Magnification:600×. (D-F) The number of LDs per cell. Compared with the NC group, the number of LDs in the IH group were obviously increased. However, treated with 3-FP could not reduce the number of LDs. (G, H) Quantitative real-time PCR experiments showed the expression levels of PDP1 and PDHA1 in Ht22 cells. The results showed no statistically difference among four groups. (I-L) The PDP1/PDHA1 pathway was activated by Western blot, and no statistically difference among four groups. (M) The activity of PDC and no significant difference could be found. All experiments were repeated three times. Data are expressed as the mean ± SEM. Statistical analysis was performed through two-way ANOVA. The treated samples were different from the controls at p < 0.05.
